# Potential of C1QTNF1-AS1 regulation in human hepatocellular carcinoma

**DOI:** 10.1007/s11010-019-03569-w

**Published:** 2019-06-06

**Authors:** Weijie Han, Guofeng Yu, Xianmei Meng, Hong Hong, Liansheng Zheng, Xiaobo Wu, Dongsheng Zhang, Boshi Yan, Yongqiang Ma, Xiaolong Li, Qiuhong Wang

**Affiliations:** 1 Department of Digestive Minimally Invasive Surgery, The Second Affiliated Hospital of Baotou Medical College, Baotou, 014030 Neimenggu China; 2 General Surgery, Suzhou Integrative Traditional Chinese and Western Medicine Hospital, Suzhou, 215101 Jiangsu China; 3 Nursing Department, The Second Affiliated Hospital of Baotou Medical College, Baotou, 014030 Neimenggu China

**Keywords:** C1QTNF1-AS1, miR-221-3p, *SOCS3*, Hepatocellular carcinoma

## Abstract

The aim of our study is to explore the regulation of C1QTNF1-AS1 on its target miR-221-3p/*SOCS3* in human hepatocellular carcinoma (HCC). To explore the underlying molecular regulation of non-coding RNA for HCC, differentially expressed patterns of lncRNAs and genes were examined by RNA-seq. GO and KEGG pathway analysis were done based on the function of mRNAs that mediated by differentially expressed lncRNAs. RT-qPCR and western blot were conducted to detect the mRNA and protein level expression of C1QTNF1-AS1, miR-221-3p, *SOCS3* and key proteins in JAK/STAT signaling pathway in HCC tissues and cells. The target miRNA of differentially expressed C1QTNF1-AS1 and *SOCS3* was miR-221-3p predicted by bioinformatics analysis. C1QTNF1-AS1 and *SOCS3* was downregulated and miR-221-3p was upregulated in HCC tissues and cells. In HepG2 and Huh-7 cells, the overexpression of C1QTNF1-AS1 or *SOCS3*, and silencing of miR-221-3p inhibited proliferation, migration, invasion and JAK/STAT signaling pathway, while promoted cell apoptosis. The results of dual-luciferase assay indicated that C1QTNF1-AS1 regulated miR-221-3p and miR-221-3p targeted *SOCS3* by directly binding. And the growth of HCC in vivo was impeded when C1QTNF1-AS1 was upregulated. Overexpression of C1QTNF1-AS1 could downregulate miR-221-3p thereby inhibited the proliferation, migration and invasion of HCC cells.

## Introduction

As one of the ten most common human malignancies and the third major cause of cancer-related death rate worldwide, hepatocellular carcinoma (HCC) has increasing global incidence and poor prognosis [1]. Despite treatments for patients with HCC are common now, the recurrence and metastasis still make the 5-year survival rates remain unsatisfied. Thus, it is urgent for scientists to explore the precise mechanisms underlying liver carcinogenesis and identify new molecular targets affecting tumor growth to develop new therapeutic strategies [2]. Epigenetic alteration is now developed to be a critical factor for cancer development. The dysregulation of lncRNAs and miRNA on protein-coding genes and their associated signaling pathways is one important part of epigenetic alterations in HCC, and is still largely unexplored [3].

Long noncoding RNAs (lncRNAs) are a subtype of noncoding RNAs longer than 200 nucleotides with 50 capped and 30 polyadenylated, lack of potential to encode protein. LncRNAs are involved in various biological processes, by regulating gene expression in *cis* or in *trans* [4]. In cancer, lncRNAs were always deregulated. Thus, elucidating of the lncRNAs function molecular mechanisms will be important for developing new strategies for cancer diagnosis and treatment [5]. The function of lncRNAs are diverse, they could regulate gene expression in epigenetic, transcriptional, posttranscriptional, and translational level. Research had proved that lncRNA-UCA1 could inhibit the miR-216b to promote the progression of HCC through activating FGFR1/ERK signaling pathway. That is to say, the lncRNAs may act as competing endogenous RNAs (ceRNAs) which downregulate miRNAs expression and then modulating their targets to affect cancer development [6]. Another study in HCC indicated that lncRNA-ATB was competitively binding with miR-200 family to upregulate ZEB1 and ZEB2 and then induced Epithelial–Mesenchymal Transition and invasion in HCC [7]. Yet, the regulation of ncRNA C1QTNF1-AS1 in HCC is still not clear. Previous studies demonstrated that by regulating tumor suppressor genes or oncogenes, miRNAs play crucial roles in many cellular biological processes, especially in tumor progression [8]. In a study about HCC, scientists revealed that miR-21 was aberrantly expressed in HCC tissues, and could increase migration and invasion of side population (SP) cells by directly targeting PTEN, RECK and PDCD4 [9]. As a member of anti-angiogenic gene-regulating miRNAs family, miR221-3p is encoded by a gene cluster on the X chromosome. MiR-221-3p was proved to initiate changes inproliferation, migration, invasion and apoptosis of a variety of human malignancy cells. Such as a research reported that miR-221-3p could enhance cell proliferation and impede cell apoptosis in pancreatic cancer, suggesting that miR-2213p could be a novel potential candidate for PCa [10]. However, studies about miR-221-3p are absent in HCC.

JAK and STAT are crucial members in JAK/STAT signaling pathway. Cytokines and growth factors activate firstly JAK and then STAT to trans-activate target genes, which determine immune reaction, cell growth and differentiation. Thus, SOCS-3 could terminate the signal transduction inJAK/STAT signaling pathway and then affect tumor progression [11]. SOCS-3 was often aberrantly inactivated in tumor tissues. In HCC, SOCS-3 was characterized to be methylation-associated silenced and resulted in enhanced cell growth and migration by restructing STAT activities in HCC cells according to a recent scientific research [12]. SOCS3 could also be modulated by miRNA as a target gene. A study found that SOCS3 could be a target of miR-455-5p. Wang et al. reported that miR-455-5p promoted the development and metastasis of non-small cell lung cancer by inhibiting SOCS3. [13]. Another study proved that in the hepatitis virus associated HCC, miR-221 targeted SOCS1 and SOCS3 to enhance the IFN’s influence on inhibiting HCV replication [14]. The relationship between miR-221-3p and SOCS3 in HCC progression has not been studied yet. In this present study, we identified C1QTNF1-AS1, miR-221-3p and SOCS3 that are altered in expression in HCC and their targeting regulatory relations using bioinformatics analysis. By biological experiments, we proved that C1QTNF1-AS1 could regulate miR-221-3p/SOCS3 axis to affect JAK/STAT signaling pathway and then finally change the cell behavior and tumor growth of HCC.

## Materials and methods

### Bioinformatics analysis

RNA-seq analysis was conducted to analyze the differential expressed lncRNAs and genes in normal and HCC tissues. The RNA data were downloaded from NCBI GEO DataSets (https://www.ncbi.nlm.nih.gov/gds/), and lncRNAs and genes expression in normal and HCC tissues were obtained after quantification and background correction. We used KEGG Orthology Based Annotation System software to detect the statistical enrichment of the candidate target genes in KEGG pathways. The co-expression network was built after calculating the correlation coefficient between differentially expressed lncRNAs and genes.

### Cells and tissues collection

*Cells* The immortalized human liver cell line MIHA and hepatocellular carcinoma cell lines HepG2 and Huh7 were obtained from American Type Culture Collection. All three cell lines were cultured in Dulbeco’s Modified Eagle’s medium (DMEM, HYCLONE), which contains 10% fetal bovine serum (FBS) and antibiotics composed of 100 U/ml penicillin and 100 mg/ml streptomycin, under a 5% CO_2_ atmosphere at 37 °C.

*Tissues* Eleven cases of HCC and eleven paired adjacent tissue samples were obtained from Baotou Medical College, China. Informed consents were collected from patients to approve the utilization of their tissues for research purposes. The ethical committee of Baotou Medical College approved the study.

### Plasmid construction and cell transfection

Negative control (NC) vector, si-C1QTNF1-AS1-1 (siRNA1), si-C1QTNF1-AS1-2 (siRNA2), pcDNA-C1QTNF1-AS1-wt, pcDNA-C1QTNF1-AS1-mut, miR-221-3p mimics, miR-221-3p mimics + pcDNA-C1QTNF1-AS1, pcDNA-*SOCS3*-wt, pcDNA-*SOCS3*-mut, si-*SOCS3*-1 (siRNA3), si-*SOCS3* -2 (siRNA4) and si-*SOCS3 *+ miR-221-3p inhibitor were transfected into HepG2 cell line, while negative control (NC) vector, pcDNA-negative control (pcDNA-NC), pcDNA-C1QTNF1-AS1-wt, pcDNA-C1QTNF1-AS1-mut, miR-221-3p inhibitor, miR-221-3p inhibitor + si-C1QTNF1-AS1, pcDNA-*SOCS3*-wt, pcDNA-*SOCS3*-mut, pcDNA-*SOCS3 *+ miR-221-3p mimics were transfected into Huh-7 cell line. All plasmids were designed and purchased from GenePharma (Shanghai, China). 24 h prior to transfection, HepG2 and Huh-7 cells were suspended again in DMEM and inoculated in 6-well culture plates for 18-24 h under 5% CO_2_ at 37 °C, to achieve 80% confluence per well. Three hours before transfection, the cell culture media were replaced into serum and antibiotic-free media. Using Lipofectamine 2000 reagent (Life Technologies, MD, USA), plasmids were transfected and incubated under the same condition as before. After 8 h, complete medium was added and cells were incubated for further analysis. Selection medium (600 mg/ml G418) was applied for routinely maintaining positive transfected clones.

### Real-time quantitative polymerase chain reaction (RT-qPCR) assay

Total RNA was extracted from tissue samples and cell lines using an RNAiso kit (Takara, China) according to the manufacturer’s instructions. The by NanoDrop 2000 (Thermo Fisher Scientific Inc, MA, USA) performed total RNA quantification. We used the 2-method to quantify the relative expression levels of mRNA and GAPDH for normalization. All of the experiments were performed in triplets.

### MTT assay

HepG2 and Huh-7 cells were suspended in complete media and then seeded in 96-well plates at 2 × 10^4^ cells/ml. The optical density (OD) value of each sample was detected at a wavelength of 490 nm using a scientific microplate reader (Bio-Rad Laboratories, CA, USA). All of the experiments were repeated three times.

### Transwell assay

According to the manufacturer’s instructions, cell migration and invasion were examined by Transwell chamber assay (8 µm; Millipore, USA). After culturing at 37 °C for 24 h, cells on the upper surface were washed with PBS twice and scraped with a cotton swab, while the invaded and migrated cells in the lower surface were washed and fixed with 4% formaldehyde for 30 min, followed by 1% crystal violet staining. Images of migrating and invading cells were taken using an inverted fluorescence microscope (Nikon, Tokyo, Japan). Each experiment was repeated for three times.

### Flow cytometry

Cell apoptosis was analyzed using PE Annexin V Kit (BD, CA, USA), according to the manufacturer’s instruction. Cells were first collected, washed twice with cold phosphate-buffered saline (PBS), and then cultured in 5 µL Annexin V-FITC for 15 min without light at room temperature. The FACS Calibur (BD, CA, USA) was used to detect the apoptosis. Analysis was carried out using FACS Diva (BD, CA, USA) software. Each experiment was repeated at least three times.

### Western blot

Total proteins were extracted from tissues and cells using RIPA lysis buffer (Beyotime, Shanghai, China). The protein concentration was quantified by the bicinchoninic acid (BCA) protein concentration assay Kit (Biyuntian, Beijing, China). Then, SDS-PAGE was used for protein separation. For the band density measurement, the Image J software (Version 1.48u, Bethesda, USA) was used, and β-actin was the internal control. Each experiment was repeated at least three times.

### Dual-luciferase assay

HepG2 and Huh-7 cells were digested by trypsin and were collected. Following the manual instruction, EasyPure Blood Genomic DNA Kit was used for genomic DNA extracting. After 48 h of incubation, Dual-Glo Luciferase Assay System was used to measure the relative luciferase activity. Each experiment was repeated at least three times.

### Tumor formation in nude mice

Twenty male BALB/C nude mice were used for tumor formation assay. HepG2 and Huh-7 cells transfected with si-C1QTNF1-AS1-1 and pcDNA-C1QTNF1-AS1, respectively, were injected into mice right flank (5 × 106 cells per inoculation point). Tumor size and weight were monitored every 7 days for the indicated time using a vernier caliper, the tumor volumes were calculated as length × width × width/2. Each experiment was repeated at least three times.

### Statistical analyses

All statistical analyses and diagrams were performed using GraphPad Prism 6.0 software (Version 6, CA, USA). Data are presented as the mean ± standard deviation (SD). All data came from at least three repetitions in each experiment. Comparisons of multiple groups were performed using one-way analysis of variance (ANOVA) followed by Tukey’s multiple comparison test. All differences were considered statistical significance at a *P* < 0.05.

## Results

### RNA-seq analysis of C1QTNF1-AS1 and *SOCS3* in HCC tissues

**Fig. 1:**
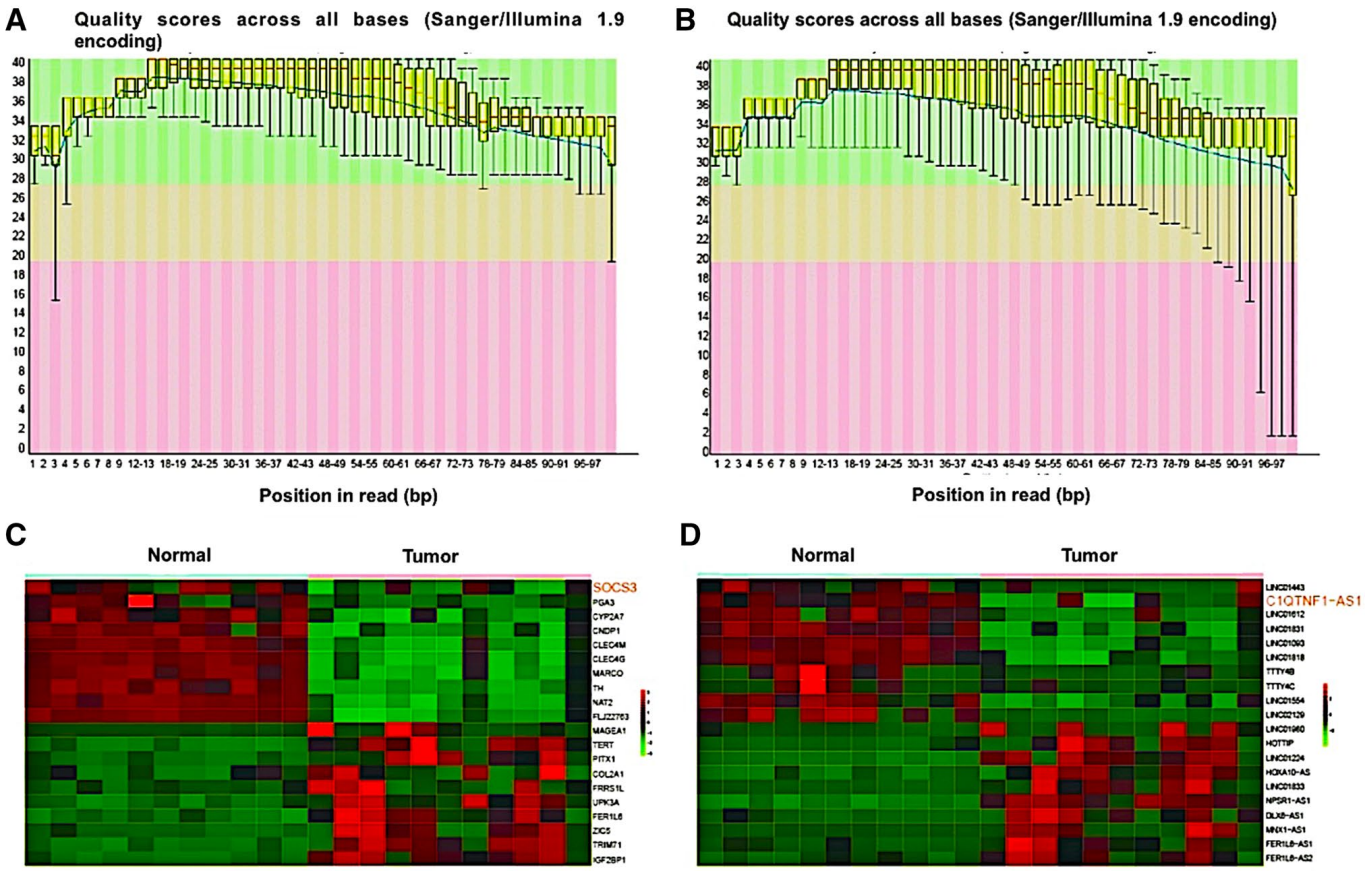
RNA-seq analysis of C1QTNF1-AS1 and SOCS3 in HCC tissues. **a**, **b** Mass fraction diagram of sample base sequences; **c** heatmap of ten most up-regulated and down-regulated mRNAs in normal and tumor tissues, SOCS3 was included in it; **d** heatmap of 10 most up-regulated and down-regulated lncRNAs in normal and tumor tissues, C1QTNF1-AS1 was included in it. (Color figure online)

The results of RNA-seq analysis of genes and lncRNAs were all under great quality control as shown in Fig. 1a, b. The green area represents highest quality scores and most bases were located in this area. By performing RNA-seq analysis, a total of 40 significantly expressed genes and lncRNAs were obtained, including 20 upregulated genes or lncRNAs and 20 downregulated ones as presented in heatmap (*P* value < 0.05, fold change ≥ 2, Fig. 1c, d). Variation analysis indicated that the expression profile of genes and lncRNAs in HCC tissues could well distinct from normal controls. Obviously, *SOCS3* and C1QTNF1-AS1 were both down-regulated in HCC tissues.

### GO function enrichment and pathway enrichment analysis

**Fig. 2:**
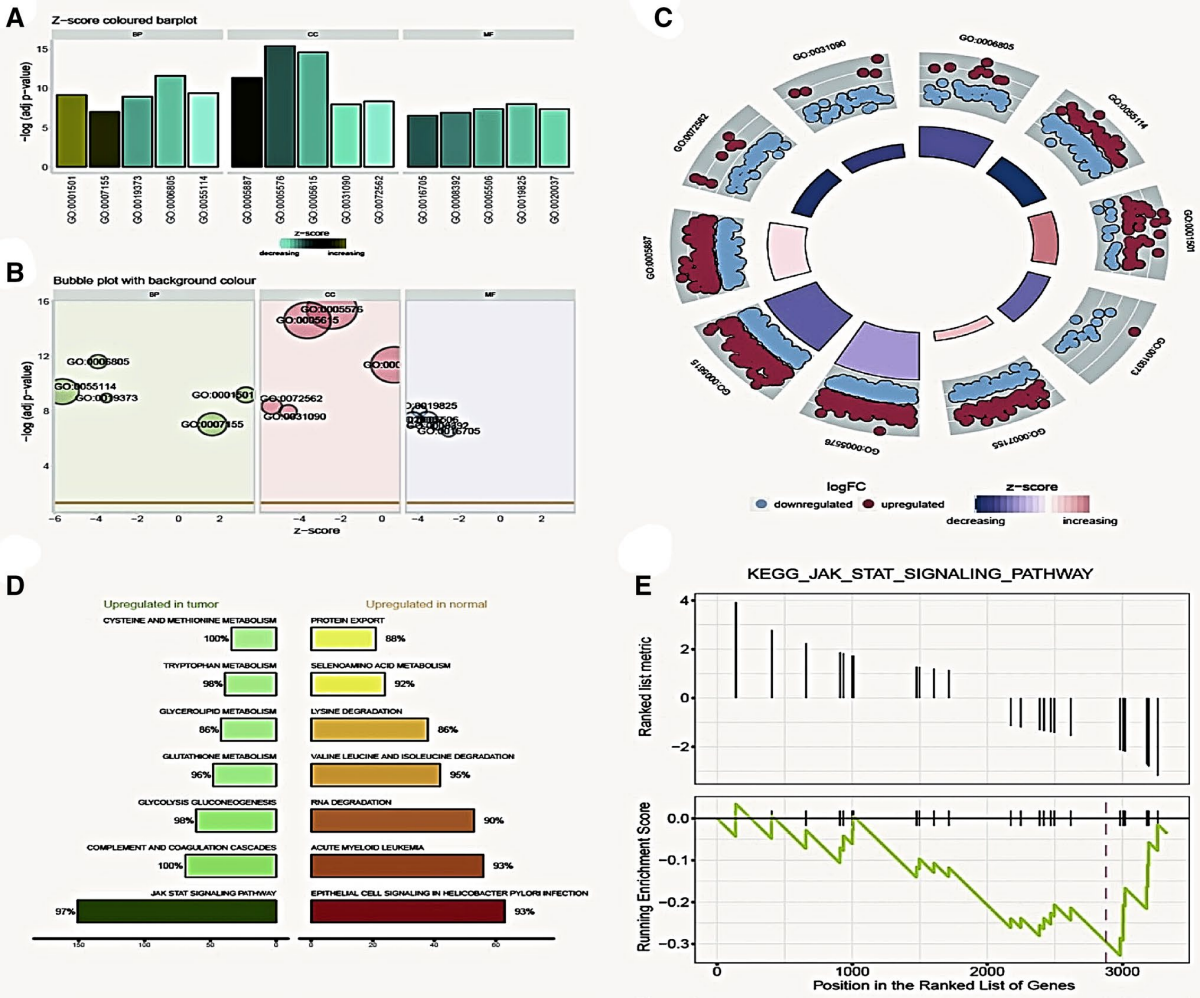
GO function enrichment and pathway enrichment analysis. **a–c** GO function analysis cluster diagram. Higher z-score presents an increase in function expression; **d** KEGG analysis revealed the different biological function in cancer and normal samples; **e** Gene enrichment in JAK/STAT pathway

We used GO plot to perform the functional analysis of differentially expressed lncRNAs and genes. Results showed the top-ranking biological processes (BPs), cellular component (CC) and molecular function (MF) they were associated with, and the most significantly related functions were xenobiotic metabolic process, integral component of plasma membrane and cell adhesion (−log_10_ adjusted *P* value, Fig. 2a–c). KEGG pathway enrichment analysis revealed the different biological functions in tumor and normal tissue samples, and that JAK/STAT signaling was upregulated one in tumor tissues (Fig. 2d, e).

### miR-221-3p is the target of C1QTNF1-AS1 and *SOCS3*

**Fig. 3:**
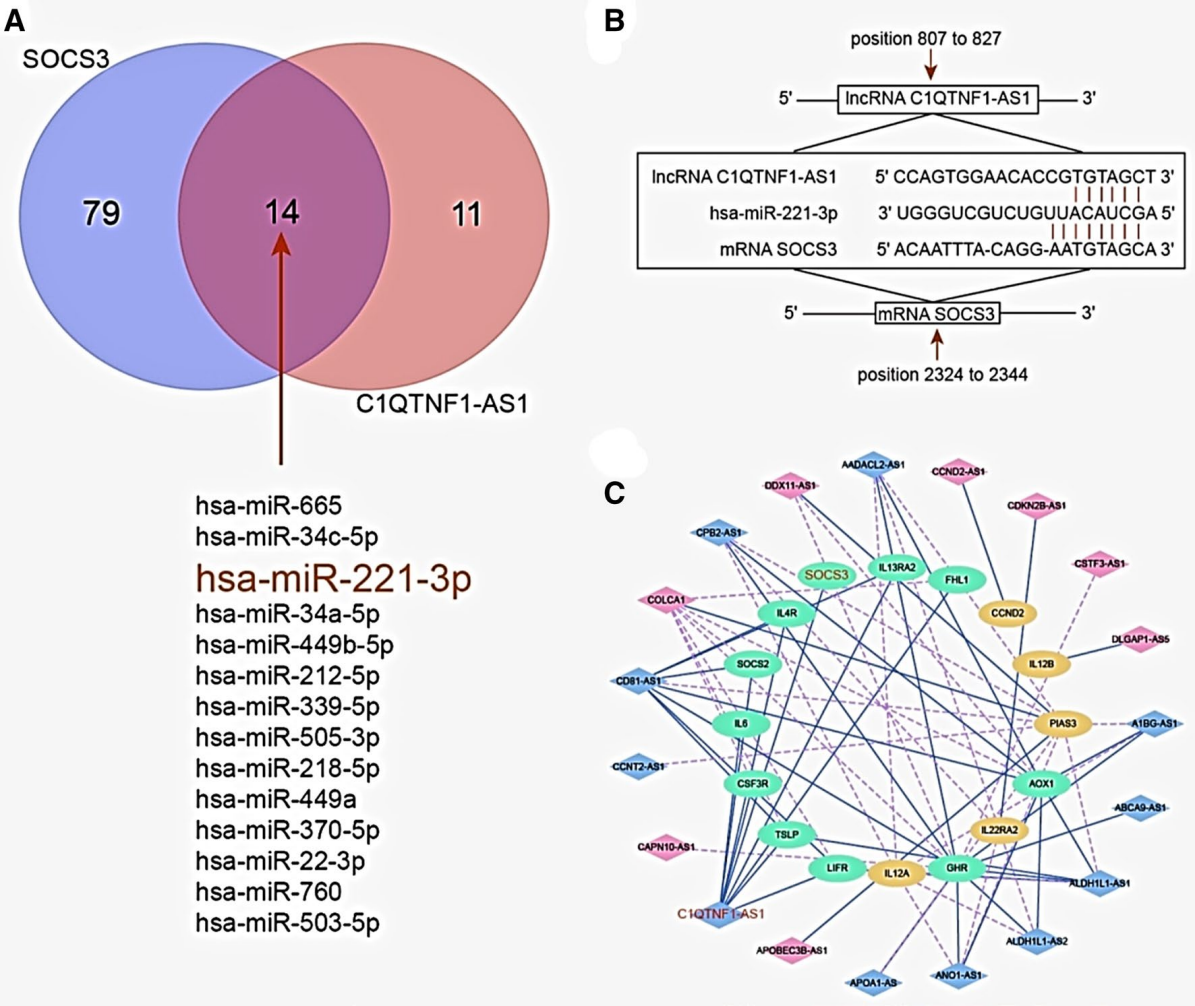
**a** MiR-221-3p is the target of C1QTNF1-AS1 and SOCS3. Prediction and screening of miRNA co-targeted by SOCS3 and C1QTNF1-AS1. MiR-221-3p is one of the targets; **b** Targeting binding site between C1QTNF1-AS1/miR-221-3p, and between SOCS3/miR-221-3p; **c** Co-expression network of mRNA and lncRNAs. C1QTNF1-AS1 and SOCS3 are included

The target miRNAs binding with C1QTNF1-AS1 and *SOCS3*, respectively, were predicted based on the analysis by TargetScan 7.1, the results were made into a intersection which includes miR-221-3p (Fig. 3a). Furthermore, the binding sites between C1QTNF1-AS1 and miR-221-3p, or *SOCS3* and miR-221-3p were shown in Fig. 3b. According to the co-expression network, *SOCS3* was demonstrated to correlate with C1QTNF1-AS1 (Fig. 3c).

### C1QTNF1-AS1 affected HCC cell behavior through JAK/STAT signaling pathway

**Fig. 4:**
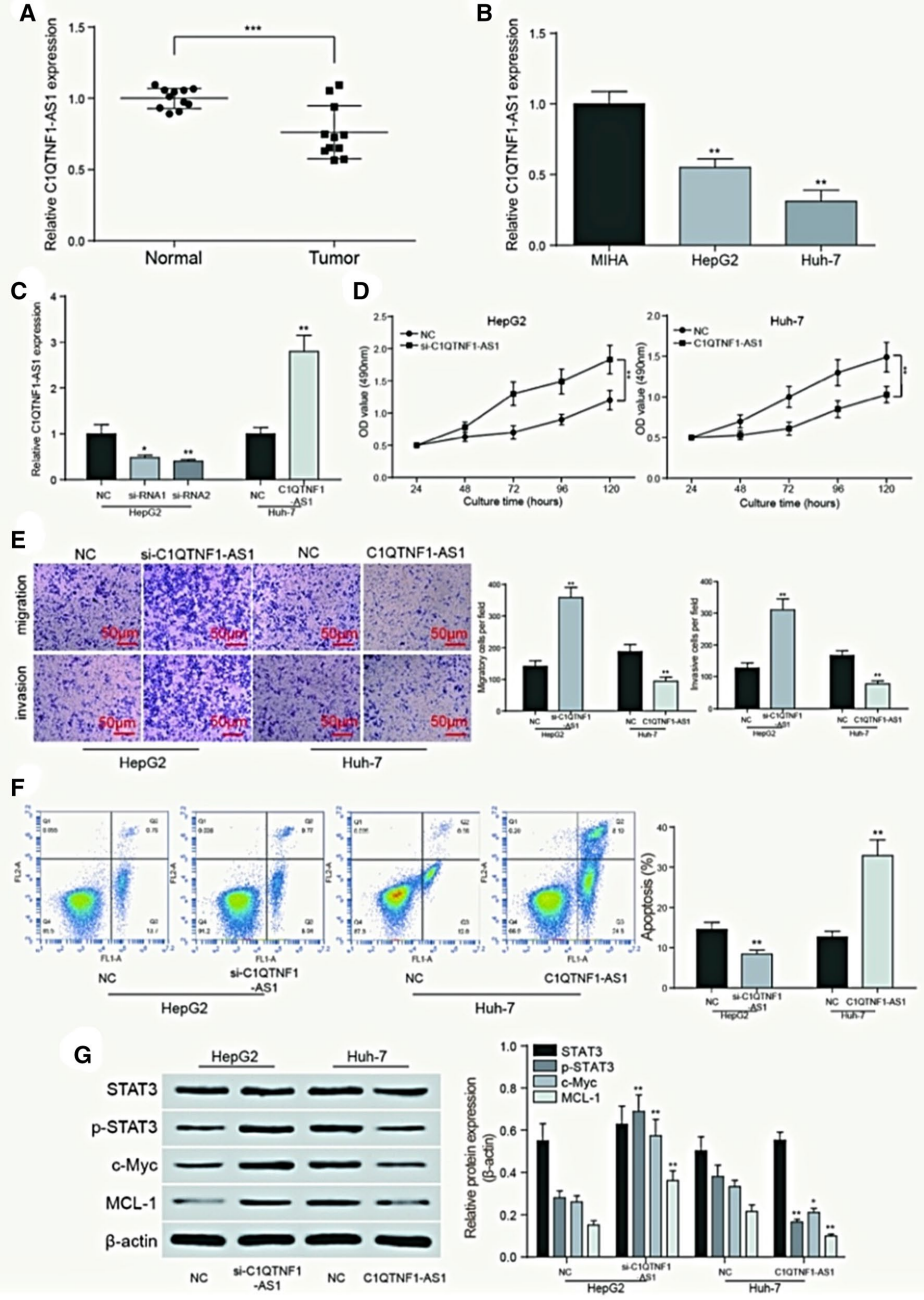
C1QTNF1-AS1 affected HCC cell behavior through JAK/STAT signaling pathway. **a** The results of qPCR showed that C1QTNF1-AS1 was low expressed in HCC tissues. **P < 0.01 compared to the normal group; **b** The expression of C1QTNF1-AS1 in cell lines HepG2 and Huh-7 were lower than that in normal human liver cell line MIHA. **P < 0.01 compared to the MIHA group; **c** test of transfection efficiency and screening of interfered RNA. **P < 0.01 compared to the NC group; **d** MTT assay showed that C1QTNF1-AS1 slowed down growth of HCC cell line Huh-7, while si-C1QTNF1-AS1 enhanced it. **P < 0.01 compared with NC group; **e** transwell assay was performed to determine the migratory and invasive capacity of HepG2 and Huh-7 cells transfected with si-C1QTNF1-AS1 and C1QTNF1-AS1, respectively. C1QTNF1-AS1 could inhibit the migration and invasion of HCC cells. **P < 0.01 compared to the NC group; **f** cells were collected and stained with Annexin V-FTIC/PI after transfection. The percentages of early (low right quadrant) and late apoptotic cells (upper right quadrant) were assessed by flow cytometry. C1QTNF1-AS1 could promote the apoptosis of HCC cells. **P < 0.01 compared to the NC group; **g** C1QTNF1-AS1 inhibits the JAK/STAT signaling pathway, which is manifested in the decline in phosphorylation of STAT, as well as the decrease in the expression of downstream proteins c-Myc and MCL-1. *P < 0.05, **P < 0.01 compared to the NC group

The expression of C1QTNF1-AS1 was detected by RT-qPCR. Compared with 11 normal tissues, the expression of C1QTNF1-AS1 was significantly lower in 11 HCC tissues (*P *< 0.001, Fig. 4a). The expression of C1QTNF1-AS1 was also detected in HCC cell lines HepG2, Huh7 and normal human liver cell line MIHA. The results suggested that C1QTNF1-AS1 expressed evidently less in HCC cells (*P *< 0.01, Fig. 4b). For transfection efficiency test and si-RNA screening, the expression of C1QTNF1-AS1 in HepG2 cells transfected with si-NC, si-C1QTNF1-AS1-1 (siRNA1), si-C1QTNF1-AS1-2 (siRNA2) and Huh7 cells transfected with pcDNA-C1QTNF1-AS1 were detected and showed in Fig. 4c. Si-RNA2 indicated better efficiency. The OD growth curve measured by MTT assay indicated that the proliferation rate was significantly higher in HepG2 cells when C1QTNF1-AS1 was inhibited (*P *< 0.01), while the proliferation rate of pcDNA-C1QTNF1-AS1 group decreased dramatically compared with NC group in Huh7 cells (*P *< 0.01, Fig. 4d). Transwell assay indicated that the invasive and migratory ability of si-C1QTNF1-AS1 group was significantly higher in HepG2 cells than that in NC group (*P *< 0.01), while the Huh7 cells with over expressed C1QTNF1-AS1 showed lower invasive and migratory ability than that of NC group (*P *< 0.01, Fig. 4e). Flow cytometry result revealed that the apoptosis rate was significantly lower than NC group when the expression of C1QTNF1-AS1 was decreased in HepG2 cells, while Huh7 cells transfected with pcDNA-C1QTNF1-AS1 presented an opposite result (*P *< 0.01, Fig. 4f). As is indicated in Fig. 4g, C1QTNF1-AS1 could restrain the JAK/STAT signaling pathway revealed by western blot. The decrease of C1QTNF1-AS1 expression in HepG2 cells led to the increase of STAT phosphorylation and the expression of downstream protein c-Myc and MCL-1, while the overexpression of C1QTNF1-AS1 in Huh7 cells resulted oppositely, compared with NC group (*P *< 0.01).

### miR-221-3p affected the HCC cell behavior and JAK/STAT signaling pathway regulated by C1QTNF1-AS1

**Fig. 5:**
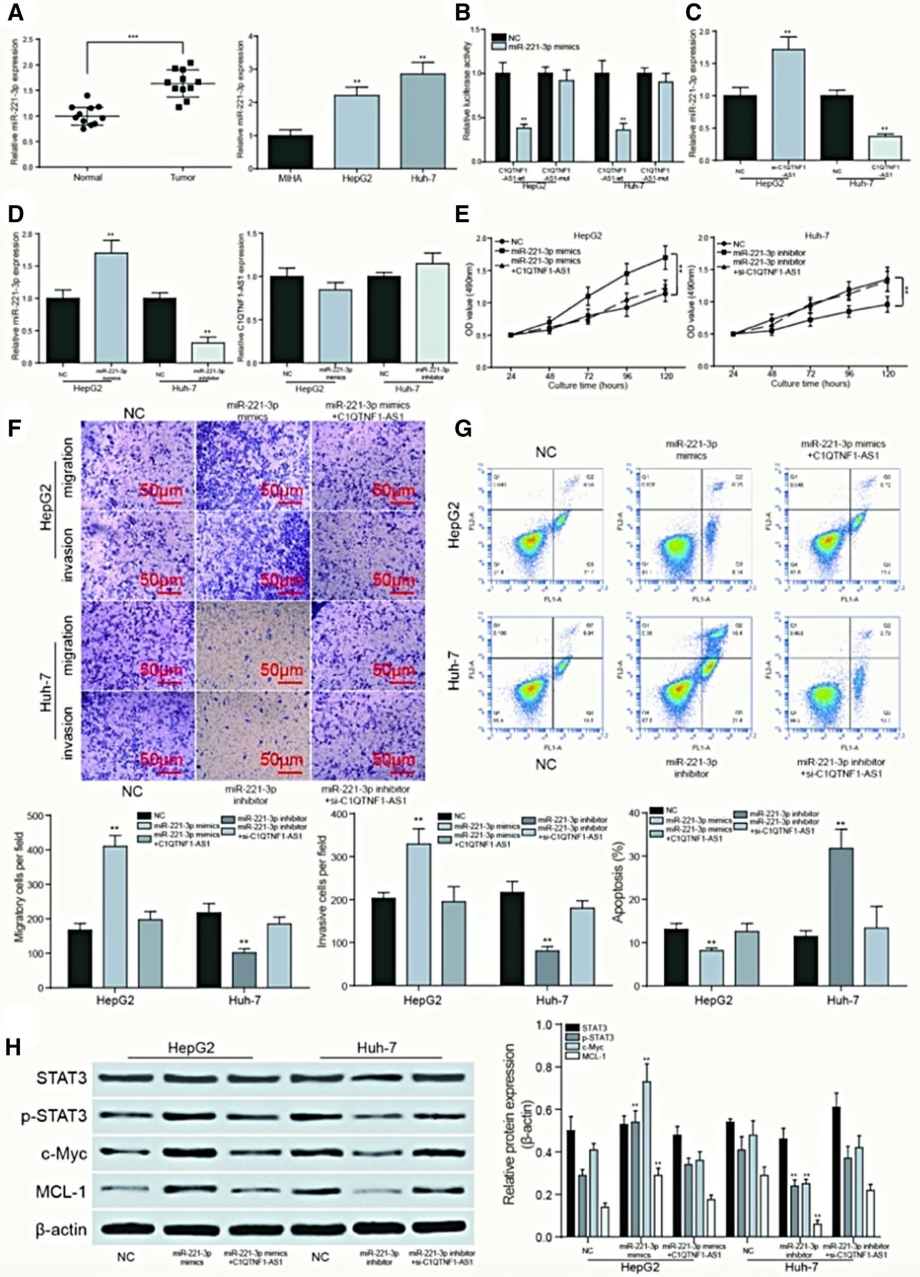
miR-221-3p affected the HCC cell behavior and JAK/STAT signaling pathway regulated by C1QTNF1-AS1. **a** The results of qPCR showed that miR-221-3p expressed more in HCC tissues. **P < 0.01 compared to the normal group; **b** dual luciferase assays confirmed the targeted binding of C1QTNF1-AS1 and miR-221-3p. **P < 0.01 compared to the NC group; **c** RT-qPCR verified that the expression of miR-221-3p was negatively regulated by C1QTNF1-AS1. **P < 0.01 compared to the NC group; **d** miR-221-3p transfection efficiency test and validation showed that the expression of miR-221-3p had no significant effect on the expression of C1QTNF1-AS1. **P < 0.01 compared to the NC group; **e** MTT assay showed that miR-221-3p promoted the growth of HCC cell line HepG2, while the addition of C1QTNF1-AS1 counteracted this effect. Inhibition of miR-221-3p could inhibit the proliferation of cancer cells, but with the inhibition of upstream C1QTNF1-AS1, the proliferation ability of cancer cells was restored. **P < 0.01 compared with NC group; **f** Transwell assay was performed to determine the migratory and invasive capacity of HepG2 and Huh-7 cells. MiR-221-3p can promote the migration and invasion of cancer cells, which is offset with the addition of C1QTNF1-AS1, and the inhibition of miR-221-3p made migration and invasion of cancer cells inhibited and recovered with the inhibition of the upstream C1QTNF1-AS1.**P < 0.01 compared to the NC group; **g** miR-221-3p can inhibit the apoptosis of cancer cells. With the addition of C1QTNF1-AS1, the inhibitory effect is offset, and the inhibition of miR-221-3p can promote the apoptosis of cancer cells, but the apoptosis of cancer cells decreases with the inhibition of the upstream C1QTNF1-AS1. **P < 0.01 compared to the NC group; **h** miR-221-3p activated the JAK/STAT signaling pathway, which was manifested in the rise of phosphorylation of STAT, and the increase in the expression of c-Myc and MCL-1, and the inhibition of miR-221-3p led to the opposite result. The above situation was regulated by upstream C1QTNF1-AS1. **P < 0.01 compared to the NC group

The expression of miR-221-3p detected by RT-qPCR was shown in Fig. 5a. In the 11 paired tissues, miR-221-3p expressed relatively higher in tumor ones (*P *< 0.001). The similar trend can be seen while the detection object was replaced by HCC cell lines HepG2, Huh7 and normal human liver cell line MIHA (*P *< 0.01). Based on the bioinformatics analysis, miR-221-3p may be a target of C1QTNF1-AS1. To verify this, HepG2 cells and Huh-7 cells were co-transfected with miR-221-3p mimics together with luciferase reporter vectors with wild-type or mutated 3′ UTR of C1QTNF1-AS1. The dual-luciferase assay showed that the addition of miR-221-3p mimics inhibited the activity of luciferase in the wild-type group, indicating that miR-221-3p could bind to the 3′-UTR seed sequence of C1QTNF1-AS1 (*P *< 0.01, Fig. 5b). The inhibition of C1QTNF1-AS1 in HepG2 cells enhanced the expression of miR-221-3p compared with NC group, while the expression of miR-221-3p in Huh7 cells transfected with pcDNA-C1QTNF1-AS1 is lower than control (*P *< 0.01, Fig. 5c). However, miR-221-3p made no difference on the expression of the upstream C1QTNF1-AS1. And the transfection of miR-221-3p mimic and miR-221-3p inhibitor in HCC cells could lead to the upregulation and downregulation of the expression of miR-221-3p (*P *< 0.01, Fig. 5d). The OD value measured by MTT assay showed that overexpression of miR-221-3p could promote the proliferation rate of HCC cells, while the addition of C1QTNF1-AS1 counteracted this effect. The inhibition of miR-221-3p and the join of si-C1QTNF1-AS1 led to the opposite result (*P *< 0.01, Fig. 5e).

Transwell assay indicated that when miR-221-3p was inhibited, the invasive and migratory ability of Huh-7 cells was significantly lower than that in NC group (*P *< 0.01), and the inhibition of C1QTNF1-AS1 at the same time could counteract the negative effect. The up-regulation of miR-221-3p and the addition of C1QTNF1-AS1 in Huh7 cells functioned in an opposite way (*P *< 0.01, Fig. 5f). Flow cytometry results revealed that the apoptosis rate was significantly lower than NC group when the expression of miR-221-3p was increased in HepG2 cells, while Huh7 cells transfected with miR-221-3p inhibitor presented an opposite result. And the joining of C1QTNF1-AS1 or si-C1QTNF1-AS1 could moderate the resulting trend (*P *< 0.01, Fig. 5g). The western blot results shown in Fig. 5h indicated that the miR-221-3p could enhance the JAK/STAT signaling pathway and the addition of C1QTNF1-AS1 counteracted this positive effect, while the suppression of miR-221-3p and C1QTNF1-AS1 triggered an opposite result. The promotion of JAK/STAT signaling pathway manifested in the increase of STAT phosphorylation and the expression of downstream protein c-Myc and MCL-1 (*P *< 0.01).

### *SOCS3* is the target gene of miR-221-3p regulated by C1QTNF1-AS1

**Fig. 6:**
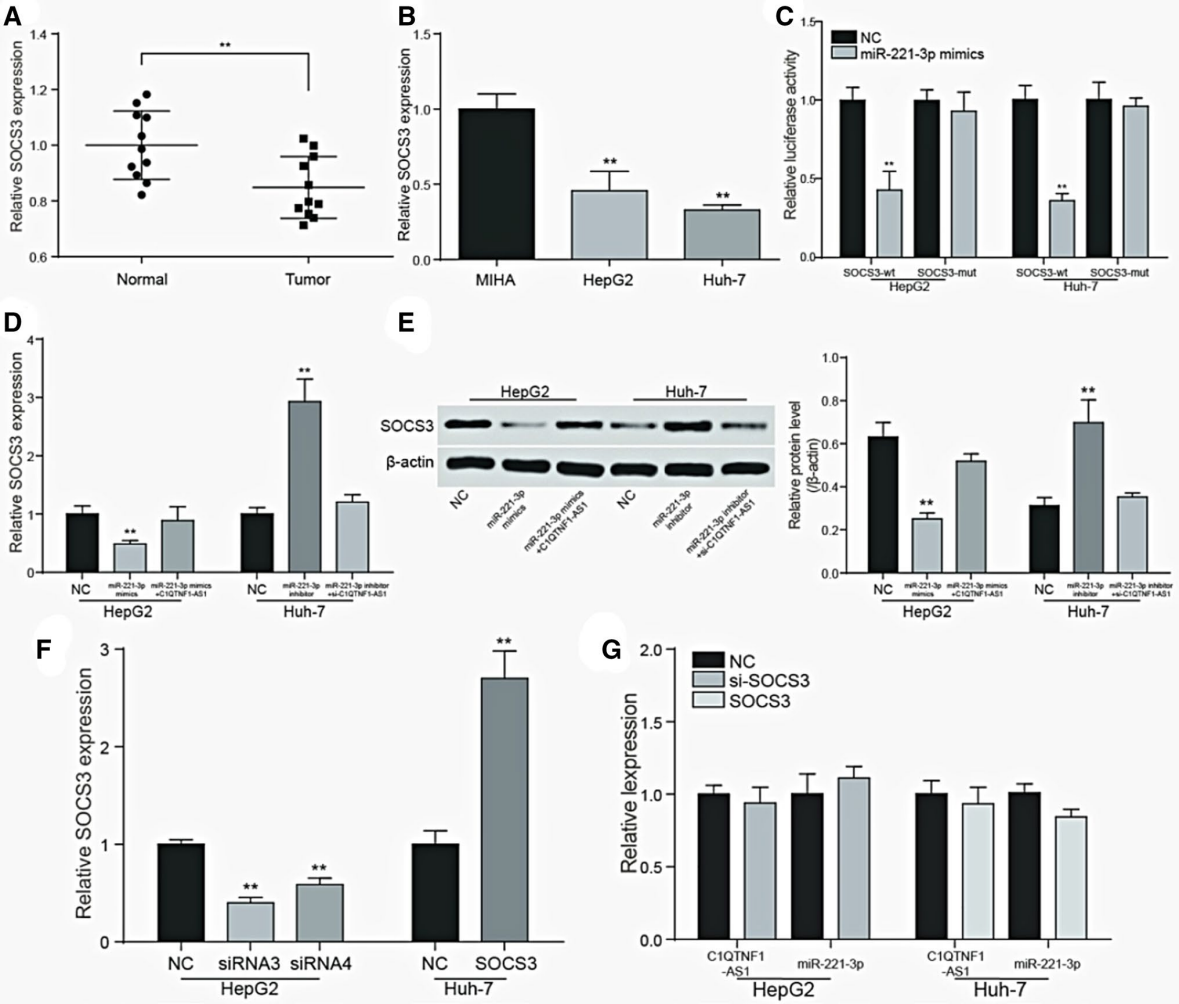
SOCS3 is the target gene of miR-221-3p regulated by C1QTNF1-AS1. **a** The results of RT-qPCR showed that SOCS3 expressed lower in HCC tissues. **P < 0.01 compared to the normal group; **b** the results of RT-qPCR showed that SOCS3 expressed lower in HCC cells. **P < 0.01 compared to the MIHA group; **c** dual luciferase assays confirmed the targeted binding of SOCS3 and miR-221-3p. **P < 0.01 compared to the NC group; **d, e** RT-qPCR and western blot results showed that the expression of SOCS3 was negatively regulated by miR-221-3p and changed with the expression of upstream C1QTNF1-AS1. **P < 0.01 compared to the NC group; **f** Test of transfection efficiency of SOCS3 and screening of interfering RNA. **P < 0.01 compared to the NC group; **g** The expression of SOCS3 had no significant effect on the upstream C1QTNF1-AS1 and miR-221-3p

The result of RT-qPCR showed the relative expression of *SOCS3*. The expression of *SOCS3* was down-regulated in HCC tissues compared with normal ones (*P *< 0.01, Fig. 6a). When it comes to the cell lines, *SOCS3* in HCC cell lines also expressed more than in normal human liver cell line MIHA (*P* < 0.01, Fig. 6b). To verify whether *SOCS3* is the target of miR-221-3p, HepG2 cells and Huh-7 cells were co-transfected with miR-221-3p mimics together with luciferase reporter vectors with wild-type or mutated 3′ UTR of *SOCS3*. Figure 6c showed that miR-221-3p mimics inhibited the activity of luciferase in the wild-type group, indicating that miR-221-3p could bind to the 3′-UTR seed sequence of *SOCS3* (*P *< 0.01). The results of RT-qPCR and western blot revealed the regulation relationship among *SOCS3*, miR-221-3p and C1QTNF1-AS1 in HepG2 cells and Huh-7 cells. The upregulation of miR-221-3p inhibited the expression of *SOCS3*, but the addition of C1QTNF1-AS1 dragged the low level expression back. And the inhibition of miR-221-3p and C1QTNF1-AS1 led to a completely contrary result, indicated that *SOCS3* is the target gene of miR-221-3p regulated by C1QTNF1-AS1 (*P *< 0.01, Fig. 6d, e). The transfection efficiency were tested by evaluating the expression of *SOCS3* after transfection of si-*SOCS3*-1 (siRNA3), si-*SOCS3* -2 (siRNA4) and pcDNA-*SOCS3* in HCC cells (*P *< 0.01, Fig. 6f). Si-RNA3 seemed to own a more obvious effect and was selected for further experiments. However, the expression of *SOCS3* had no influence on C1QTNF1-AS1 and miR-221-3p according to Fig. 6g, since there was no significant change of C1QTNF1-AS1 and miR-221-3p after transfection with si-*SOCS3* and pcDNA-*SOCS3* in HepG2 and Huh-7 cells.

### *SOCS3* affected the HCC cell behavior and JAK/STAT signaling pathway regulated by miR-221-3p

**Fig. 7:**
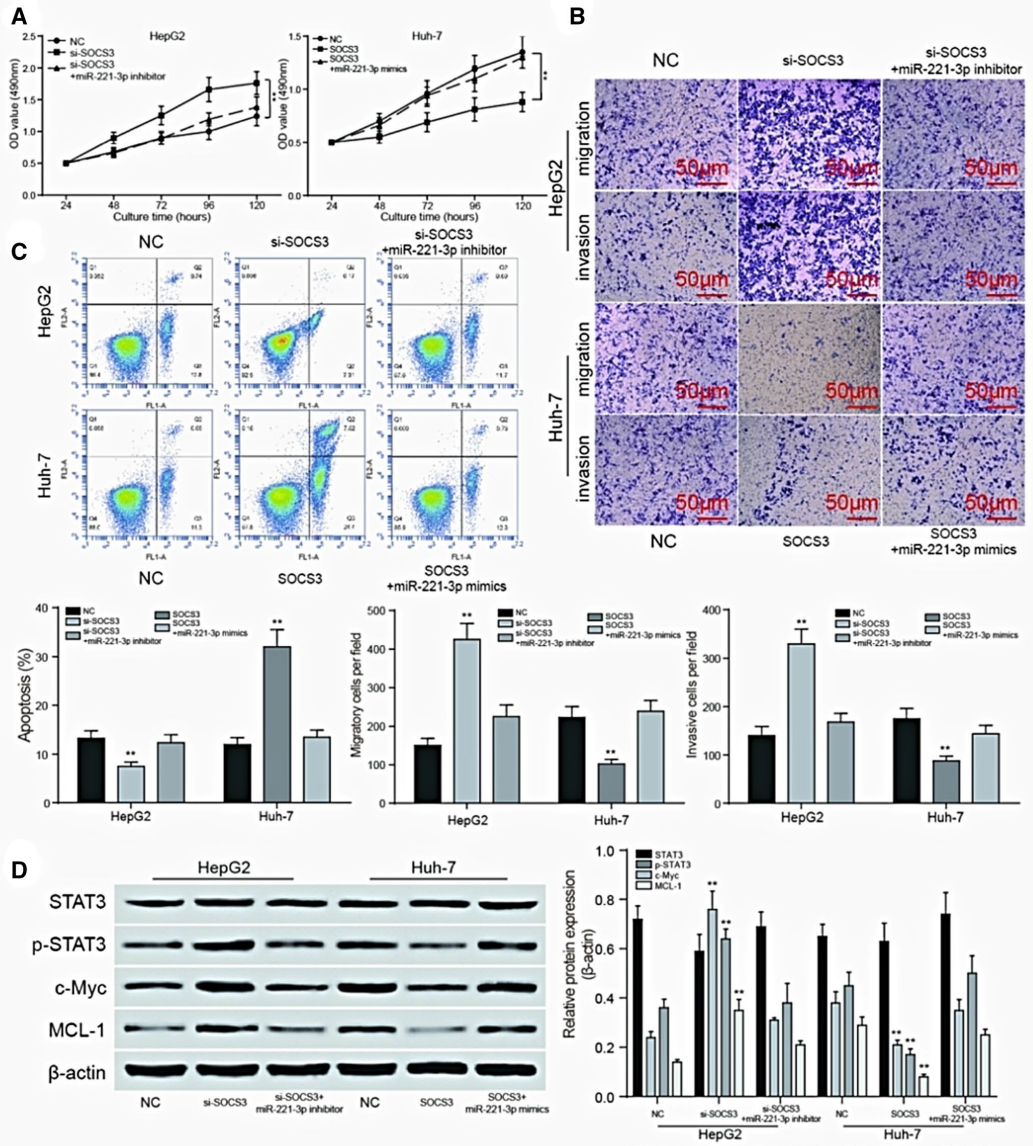
SOCS3 affected the HCC cell behavior and JAK/STAT signaling pathway regulated by miR-221-3p. **a** MTT assay showed that SOCS3 can inhibit the proliferation of cancer cells. With the addition of miR-221-3p, the inhibitory effect is offset, and the inhibition of SOCS3 can promote the proliferation of cancer cells, but the proliferation ability of the cancer cells decreases with the inhibition of the upstream miR-221-3p. **P < 0.01 compared with NC group; **b** Transwell assay was performed to determine the migratory and invasive capacity of HepG2 and Huh-7 cells. SOCS3 can inhibit the migration and invasion of cancer cells. With the addition of miR-221-3p, the inhibitory effect is offset, and the inhibition of SOCS3 can promote the migration and invasion of cancer cells, but the migration and invasion ability of cancer cells decreases with the inhibition of the upstream miR-221-3p.**P < 0.01 compared to the NC group; **c** SOCS3 can promote the apoptosis of cancer cells. With the addition of miR-221-3p, the promotion effect is offset, and the inhibition of SOCS3 can inhibit the apoptosis of cancer cells, but the apoptosis of cancer cells increases with the inhibition of the upstream miR-221-3p. **P < 0.01 compared to the NC group; **d** SOCS3 inhibits the JAK/STAT signaling pathway, which is manifested in the decline in phosphorylation of STAT, as well as the decrease in the expression of c-Myc and MCL-1 in the downstream proteins, and the inhibition of SOCS3 will lead to the opposite result. And the above situation will change with the regulation of upstream miR-221-3p. **P < 0.01 compared to the NC group

The OD value measured by MTT assay showed that upregulation of *SOCS3* could inhibit the proliferation rate of HCC cells, but as the upstream miR-221-3p is suppressed, the proliferation ability of cancer cells decreased. The raise of *SOCS3* and the join of miR-221-3p made an opposite result (*P *< 0.01, Fig. 7a). Transwell assay showed that when *SOCS3* was inhibited, the invasive and migratory rate of HepG2 cells was significantly higher than that in NC group (*P *< 0.01), and the inhibition of miR-221-3p at the same time could counteract the increase trend. The upregulation of *SOCS3* and the addition of miR-221-3p in Huh7 cells functioned in an opposite way (*P *< 0.01, Fig. 7b). Flow cytometry results showed that the apoptosis rate was significantly lower than NC group when the expression of *SOCS3* was decreased in HepG2 cells, while Huh7 cells transfected with pcDNA-*SOCS3* presented an opposite result. And the up or downregulation of miR-221-3p could moderated the above results (*P *< 0.01, Fig. 7c). The western blot results shown in Fig. 7d indicated that the *SOCS3* could inhibit the JAK/STAT signaling pathway and the co-transfection with miR-221-3p mimic counteracted this negative effect, while the inhibition of *SOCS3* and miR-221-3p led to an opposite result. The decrease of STAT phosphorylation and the expression of downstream protein c-Myc and MCL-1 characterized the inhibition of JAK/STAT signaling pathway (*P *< 0.01).

### The overexpression of C1QTNF1-AS1 inhibited tumor growth

**Fig. 8:**
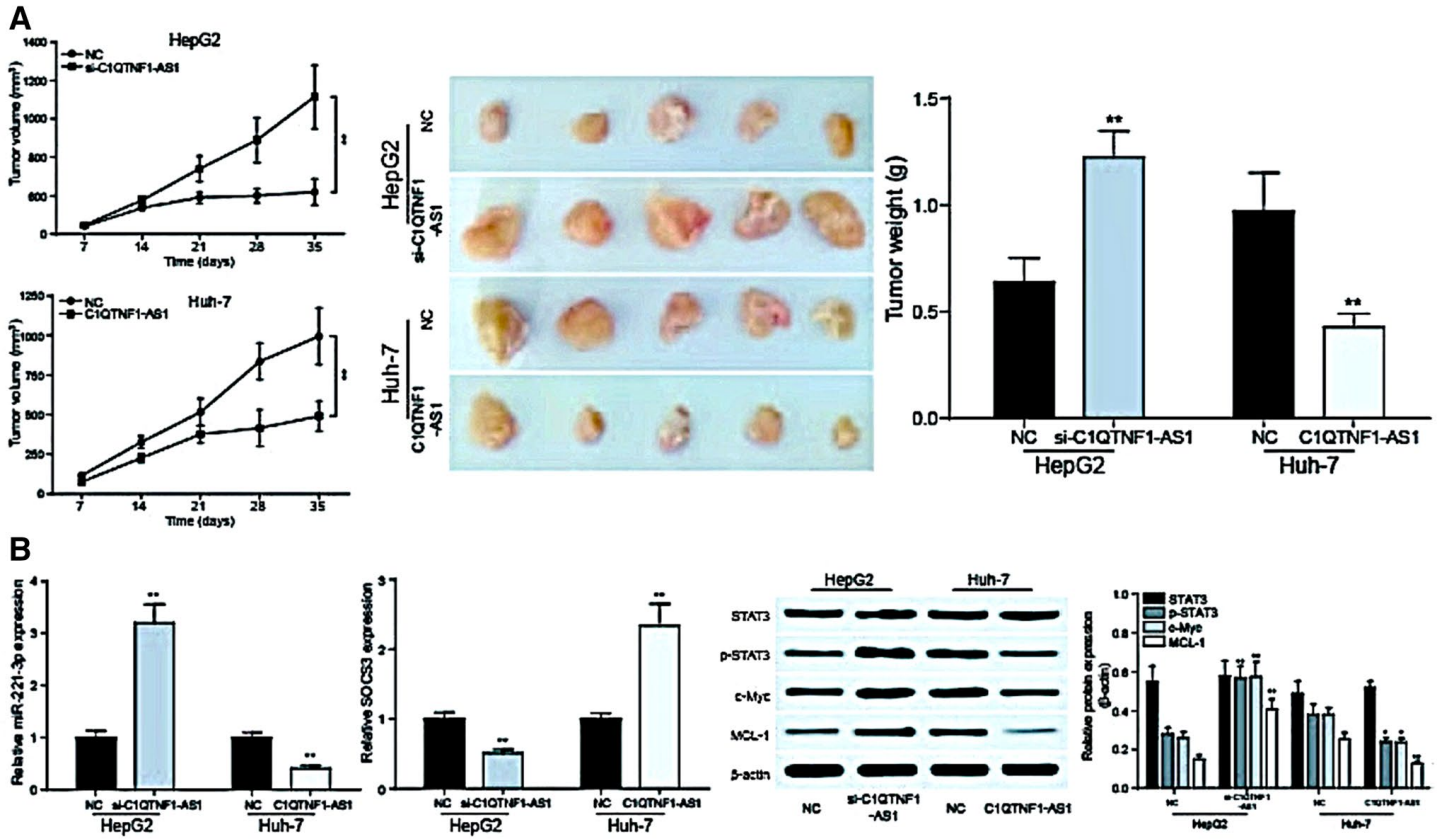
The overexpression of C1QTNF1-AS1 inhibited tumor growth. **a** Tumors were collected from nude mice injected with HCC cells transfected with si-C1QTNF1-AS1 or C1QTNF1-AS1 or NC. The overexpression of C1QTNF1-AS1 inhibits the growth of the tumor, and the inhibition of the expression of C1QTNF1-AS1 promoted the formation of the tumor. **P < 0.01 compared to the NC group; **b** expression of miR-221-3p, SOCS3 and JAK/STAT signaling pathway proteins in nude mice tumorigenicity assay. **P < 0.01 compared to the NC group

The overexpression of C1QTNF1-AS1 dramatically inhibited the growth of hepatocellular carcinoma tumor in nude mice in contrast with the control group. As for the tumor volume, tumors in C1QTNF1-AS1 group were dramatically smaller than that of NC group. At the 35^th^ day, the tumors were collected from the nude mice and weighted with electronic balance. The result of this showed that the upregulation of C1QTNF1-AS1 also made the tumors lighter than NC group (Fig. 8a, *P *< 0.01). After tumor formation in nude mice, the expression of miR-221-3p, *SOCS3* and proteins in JAK/STAT signaling pathway were detected by RT-qPCR and western blot. In the tumor tissues formed by HepG2 cells transfected with si-C1QTNF1-AS1, the expression of miR-221-3p, *SOCS3*, p-STAT3, c-Myc and MCL-1 was increased, decreased, increased, increased and increased, respectively. And in tumor tissues formed by Huh-7 cells transfected with pcDNA-C1QTNF1-AS1, results were opposite (Fig. 8b, *P *< 0.01).

## Discussion

Over the past 20 years, mortality of HCC has increased consistently. Despite advanced diagnosis and treatments like liver resection, liver transplantation and radiofrequency ablation have been developed, the prognosis of HCC is still very poor [15]. Since the process of hepatic carcinogenesis has always been described involving a range of genetic alterations, the key roles of recently identified lncRNAs, miRNAs and related protein-coding genes in hepatic carcinogenesis now could be inspiration of developing new diagnosis and treatment strategies and improving the overall prognosis of HCC patients [16]. In our present study, we investigated the differential expressed lncRNAs and genes between HCC tissues and normal tissues, and predicted their target miRNAs using bioinformatics analysis. LncRNA C1QTNF1-AS1 and gene *SOCS3* were identified to be both down-regulated in HCC tissues, while their target miR-221-3p was upregulated. We found that C1QTNF1-AS1 could inhibit proliferation, migration, invasion and JAK/STAT signaling pathway and promote apoptosis in HCC cells by suppressing miR-221-3p and fatherly promoting *SOCS3*. The novel C1QTNF1-AS1/miR-221-3p/*SOCS3* regulatory axis was elucidated in HCC tissues and cells.

LncRNAs has caught much attention since they were first discovered. They were always involved in gene expression at epigenetics as well as carcinogenesis. Previous studies proved that lncRNAs were important regulators in tumorigenesis and subsequent prognosis and metastasis of malignances [17]. Such as Han et al. have reported that the down regulated lncRNA GAS6-AS1 could affect the development and progression of non-small cell lung cancer and thus became the potential candidate of diagnostic target for non-small cell lung cancer [18]. LncRNA C1QTNF1-AS1 was first identified by RNA-seq carried out by Fagerberg et al. [19]. However, scientific research about C1QTNF1-AS1 is still absent now. In our research, we identified downregulation of C1QTNF1-AS1 in HCC tissues for the first time. By applying gain-of-function approach, we demonstrated that promoted expression of C1QTNF1-AS1 could restrain the proliferation, migration and invasion and enhance the apoptosis of HCC cells by suppressing miR-221-3p, which mains the deregulation of C1QTNF1-AS1 may affect HCC progression by acting as sponges for miR-221-3p. As we first discovered the low expression of C1QTNF1-AS1 in HCC tissues and elucidated its regulatory axis in HCC, a new potential therapeutic targets for HCC has been born. Further study may be urgently in need to apply C1QTNF1-AS1 to the development of new clinical diagnosis and treatment methods.

One of the most critical lncRNAs’ regulation mechanisms is that it can act as ceRNAs to suppress miRNA and further affect the target gene of miRNA. And then miRNA would modulate gene expression and participate in the development and progression of different tumors [20]. In our investigation, the target miRNA of C1QTNF1-AS1 identified by bioinformatics analysis and dual-luciferase assay was miR-221-3p. Previous studies have reported that miR-221-3p could be involved in many cancer cell malignant behaviors, such as proliferation, apoptosis, invasion, metastasis, and chemoresistance. There is an example that elaborates the key role of miR-221-3p in chemoresistance. In 5-FU resistant pancreatic cancer cells, the overexpression of miR-221-3p promoted the cell proliferation, migration, invasion, and epithelial-mesenchymal transition (EMT) by inhibiting RB1 expression [21]. In our study, we detected the expression of miR-221-3p in HCC tissues and adjacent normal tissues. The result showed that the expression of miR-221-3p was relatively higher in HCC tissues, which suggested that miR-221-3p may be involved in HCC pathogenesis or development. Research about the role miR-221-3p played in HCC progression and metabolism has not been probed before us, but there are some studies about miR-221-3p in other cancer cells. For instance, a study about breast cancer reported by Deng et al. found out that the downregulation of miR-221-3p may contribute to the poor prognosis of TNBC patients by regulating PARP1. MiR-221-3p acted as an inhibitor of PARP1 by binding on its 3′-untranslated region [8]. As for colon cancer, by evaluating the relationship between miR-221-3p expression and clinical features and prognosis of patients, Tao et al. demonstrated that the overexpression of miR-221-3p was associated with lower survival rate. That’s to say, miR-221-3p may become a new signature for accurate prognostic evaluation [22].

Our discovery of high expression of miR-221-3p in HCC makes it possible to be a new therapeutic target and novel prognostic biomarker. We may utilize the study of miR-221-3p to improve treatment decisions and advance prognostic prediction. The effects of miRNAs are exerted by production of an RNA-inducing silencing complex (RISC) to restrain the translation of target genes, or straightly degrading it. TargetScan analysis was applied to predict SOCS3 as the potential target gene of miR-221-3p. And the downregulation of SOCS3 was tested in HCC when miR-221-3p was upregulated. Furthermore, luciferase reporter assay proved that SOCS3 could physically interact with miR-221-3p. All of the above results suggested that SOCS3 was the terminus of the lncRNA-miRNA-mRNA axis in HCC. In fact, accumulating studies have focused on the regulation of SOCS3 in cancers. In line with our report, a study by Guo et al. displayed the same low expression of SOCS3 in HCC. They further discovered that the inhibition of SOCS3 could enhance STAT3 activation to increase HCC cell proliferation, which reminds us of the application of rising SOCS3 in HCC prevention [23]. A report about the regulation of SOCS3 in pancreatic cancer cells found out that, the restoration of miRNA let-7 promoted the expression of SOCS3 and then impeded the activation of STAT3 [24]. Both of the above studies indicated the negative correlation between SOCS3 and STAT. As a member of protein suppressor of cytokine-signaling family, the expression of SOCS3 is tightly related to many signaling pathways and finally affects the protein encoding in cell. We detected some key proteins in JAK/STAT signaling pathway, the results showed that overexpression of SOCS3 could inhibit the STAT phosphorylation, which is consistent with previous studies. The mechanism of this inhibition has been reported to be that SOCS3 could act as a blocker of JAK kinase, and stops its interaction with STAT [25]. In our report, the overexpression of SOCS3 eventually led to the increase of proliferation, invasion, migration and the decrease of apoptosis in HCC cells. This may result from the suppression of JAK/STAT signaling pathway. Zhang et al. has reported that in human melanoma A375 cells, the block of JAK/STAT signaling pathway accounted for the enhancement in apoptosis and inhibition in proliferation, meeting the conclusion of our study [26]. The influence of SOCS3 on tumor progression may also occur through other signaling pathways. A research about the effect of SOCS3 on cell migration launched by Stevenson et al. indicated that, in murine embryonic fibroblasts and human 293T cells expressing SOCS3, the migration ability towards chemokine CCL11 was reduced through enhancing both FAK and RhoA activity by SOCS3 [27]. Our finding of the target gene SOCS3 and its regulation on JAK/STAT signaling pathway may provide a new thought on cure strategies for HCC.

To sum up, we found a novel C1QTNF1-AS1/miR-221-3p/*SOCS3* regulatory axis in HCC. By prediction from chip assay and identification from a series of biological experiments, we demonstrated the deregulation of C1QTNF1-AS1, miR-221-3p and SOCS3 in HCC tissues and revealed the physical binding between C1QTNF1-AS1/miR-221-3p and miR-221-3p/*SOCS3*. We also proved the effect of regulatory axis on the proliferation, invasion, migration and apoptosis of HCC cells through JAK/STAT signaling pathway. All of our findings could provide elicitation for developing better diagnosis and prognosis of HCC patients. However, there are also limitations in our study, for we only took two cell lines into consideration, and the mechanism of the effect of SOCS3 on HCC cell behavior is still not clear as we only tested one signaling pathway.
